# Influence of Seasonality and Circulating Cytokines on Serial QuantiFERON Discordances

**DOI:** 10.1155/2018/6731207

**Published:** 2018-03-12

**Authors:** Marsha L. Griffin, Saroochi Agarwal, Melissa Zhu Murphy, Larry D. Teeter, Edward A. Graviss

**Affiliations:** ^1^Department of Pathology and Genomic Medicine, Houston Methodist Research Institute, Houston, TX 77030, USA; ^2^Paul L. Foster School of Medicine, Texas Tech University Health Sciences Center, El Paso, TX 79905, USA

## Abstract

*Objectives. *An 18-month prospective study serially tested healthcare workers (HCWs) for tuberculosis infection (TBI) and reported discordant QuantiFERON Gold In-Tube® (QFT) results in some participants. The purpose of the current study was to investigate whether the interferon-gamma (IFN-*γ*) measured by QFT in discordant individuals could be influenced by other circulating cytokines that vary seasonally at the time of phlebotomy.* Methods. *The CDC funded TBESC Task Order 18 (TO18) project to assess the use of Interferon Gamma Release Assays (IGRAs), T-SPOT.*TB*® and QFT, compared to the tuberculin skin test (TST) for the serial testing of TBI in HCW at 4 US sites. Unstimulated plasma from 9 discordant TO18 participants at 4 different time points from the Houston site was multiplexed to determine the association between circulating cytokines and antigen stimulated IFN-*γ* levels.* Results. *IL-12, IL-1*β*, IL-3, GCSF, and IL-7 were associated with the amount of IFN-*γ* measured in response to antigen stimulation. In addition to these cytokines, a significant relationship was found between a positive QFT result and the spring season.* Conclusions. *Allergens during the spring season can result in the upregulation of IL-1*β* and IL-3, and this upregulation was observed with the amount of IFN-*γ* measured in discordant results.

## 1. Introduction

For over a century, the tuberculin skin test (TST) has been used to diagnose tuberculosis (TB) infection (TBI). TST detects delayed type hypersensitivity reaction to tuberculin purified protein derivative (PPD), but the TST is only moderately specific for* Mycobacterium tuberculosis (Mtb)* [1]. In the last decade, Interferon Gamma Release Assays (IGRAs) have been developed to improve the specificity of TBI detection, and IGRAs directly or indirectly measure the release of interferon-gamma (IFN-*γ*) by peripheral blood mononuclear cells (PBMCs) upon stimulation by the RD1-encoded* Mtb* specific antigens, ESAT-6, CFP-10, and TB7.7 (Rv2654c) [1]. During the timeframe of the present study, there were two diagnostic platforms for IGRAs: QuantiFERON-TB Gold In-Tube (QFT, Qiagen Inc., Germantown, USA) and T-SPOT.*TB*® (T-SPOT, Oxford Immunotec, Marlboro, USA) approved for use in the US by the Food and Drug Administration (FDA) [2]. The QFT assay uses an enzyme-linked immunosorbent assay (ELISA) technology to indirectly measure the amount of IFN-*γ* in whole blood plasma, whereas T-SPOT uses the enzyme-linked immunospot assay (ELISPOT) to measure the number of IFN-*γ* secreting T-cells in peripheral blood mononuclear cells (PBMCs) [2]. IGRA specificity for the diagnosis of TBI using the QFT and T-SPOT has been estimated to be 65.8% and 74.5%, respectively, and the sensitivities of QFT and T-SPOT are 84.0 and 84.2%, respectively [3].

Cytokines are immunoregulators that are important for inflammation, infection, and neoplastic processes. Cytokines are circulating intercellular signaling molecules that can induce each other via cascades, and increased cytokine concentrations can lead to complex systemic effects such as fever, shock, and chronic inflammation. Chemokines, a subset of cytokines, direct extracellular signaling and host immune effector cell migration through chemotaxis [4]. IFN-*γ* is an important immunomodulator that regulates immune responses to allergens and proteins including* Mtb *antigens [5]. The INF-*γ* cytokine is activated by the T_H1_ pathway and stimulates macrophages' antimicrobial activity and upregulates downstream chemokines such as IP-10 (CXCL10), MIP-1*α*, and MIG [6]. Given the complexity of cellular immune responses, in the host* mycobacteria* and the environment, IFN-*γ* production can be stimulated through a combined repertoire of multiple circulating cytokines and chemokines. A seasonal variation has been seen in healthy subjects whose whole blood production of cytokines occurred due to exposure to endotoxin (LPS) stimulation indicating that IFN-*γ* production may vary with factors that are affected by seasonality such as airborne pollens [7, 8].

In February 2008, the Centers for Disease Control and Prevention (CDC) Tuberculosis Epidemiologic Studies Consortium (TBESC) began an IGRA comparative study (Task Order 18, TO18) in healthcare workers (HCWs) whereby 2600 HCWs at 4 sites were enrolled in serial phlebotomy studies. Phlebotomy occurred at 4 time points—baseline, 6 months, 12 months, and 18 months—and QFT, T-SPOT, and TST were performed as per the study protocol on the enrolled HCWs [9, 10].

The current project evaluated banked clinical specimens from the 650 participants at the Houston (TX, USA) site of the TO18 study. Cocirculation serum factors from Houston site study participants with serially discordant (“flip-flop”) QFT results were analyzed using a 39-multiplex (39-plex) Luminex-based assay. The hypothesis for this retrospective “freezer” study was as follows: given the complexity of cellular immune responses, could the IFN-*γ* measured by the QFT in discordant individuals be influenced by other circulating cytokines that vary seasonally at the time of blood draw?

## 2. Methods

All plasma samples from the TO18 Houston site were frozen at −80°C after initial IGRA testing. Among 579 enrolled and consented HCWs that were tested at all four serial time points, 10 serum sets had discordant QFT results. Four (4) nil plasma samples (with no TB antigen stimulation) for each of the 10 HCWs were selected and thawed at 4°C, followed by centrifuging at 1000 ×g for 10 minutes prior to performing the multiplex assay.

We employed Millipore Luminex technology (Luminex Corp., Austin, TX) for a 39-plex magnetic bead arrays assay. The concentrations of the following circulation cytokines and chemokines were measured: IL-1*α*, IL-1*β*, IL-1ra, IL-2, sIL-2R*α*, IL-3, IL-4, IL-5, IL-6, IL-7, IL-8, IL-9, IL-10, IL-12(p40), IL-12(p70), IL-13, IL-15, IL-17a, sCD40L, EGF, Eotaxin, FGF-2, Flt-3 ligand, Fractalkine, G-CSF, GM-CSF, GRO, IFN*α*2, IFN-*γ*, IP-10, MCP-1, MCP-3/CCL7, MDC/CCL22, MIP-1*α*, MIP-1*β*, TGF*α*, TNF*α*, TNF*β*, and VEGF.

Multiplex assay procedures were followed according to the manufacturer's protocol. In summary, a magnetic bead-based assay was used to measure 39 cytokines and chemokines in 25 uL of specimen. Based on a standard curve constructed of 7 concentrations produced by serial dilution of a manufacturer-provided reagent, optical density values were converted to concentrations (pg/mL). Using the curve-fit measurements for each standard, we also estimated coefficients of variation (% CV) across duplicates. A minimum of 50 bead events were required for the software package (Luminex 200 xPONENT 3.1) to detect cytokines, and Luminex analysts generated were raw-data-analyzed.

For the analysis, we extrapolated samples with % CV higher than 25%. Generalized Estimating Equation (GEE) models were used to analyze the associations between QFT reported results (antigen induced IFN-*γ*) for TB antigen stimulated plasma and the circulating cofactors detected in the unstimulated plasma using the Millipore Luminex 39-plex. One of the 10 HCWs had extreme discordant results (±4 standard deviations) and was removed from the final data analysis. The GEE modeling allowed for multiple comparison controlling of the 36 results coming from 9 individuals. Univariate and multivariate analyses were conducted to assess the relationship between IFN-*γ* levels measured from antigen stimulated whole blood and the circulating factors. *P* values ≤ 0.05 were considered statistically significant.

## 3. Results

**Figure 1:**
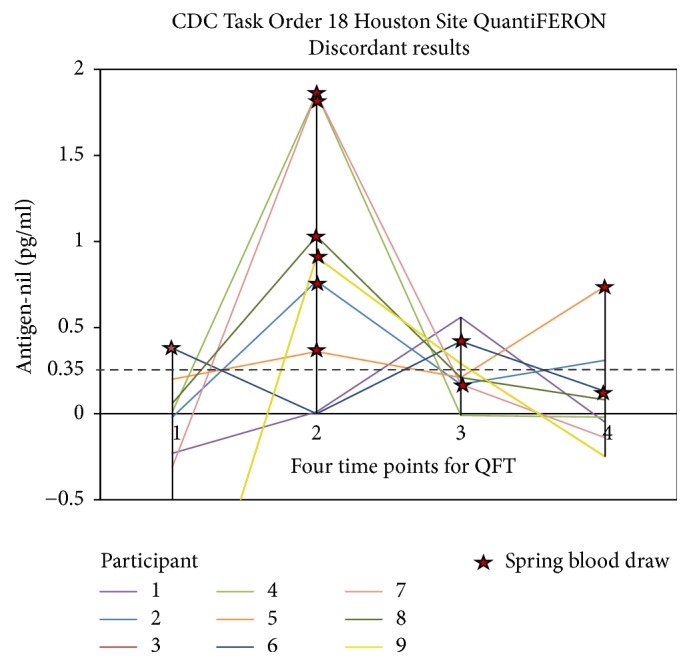
The results of the QFT of 9 out of the 28 individuals with serially discordant results are graphed here. A positive result is defined as an antigen response of 0.35 IU of IFN-*γ* or greater (after subtracting the IFN-*γ* measured in the nil QFT tube). The spring time points for each individual are identified with a star on the graph.

Among the 579 people enrolled at the Houston site with 4 results from 4 serial QFT assays, there were 9 participants whose QFT results were serially discordant (“flip-flop”) and had assay values within 2 standard deviations (Figure 1), when measured with a 39-plex Luminex-based assay. For the 36 results from the 9 individuals whose frozen specimen were analyzed using the 39-plex Luminex assay and included in the statistical analysis, the cofactors in the GEE modeling technique showing significant results were IL-7, IL-1*β*, IL-3, IL-12p70, IFN-*γ*, and granulocyte colony-stimulating factor (GCSF) (Table 1).

Among all the cofactors in the GEE modeling technique showing significance, spring season, identified as 3 months (March–May) of the year when phlebotomization occurred, had a coefficient of 33.097 that corresponds to an increase of 33 pg of IFN-*γ* produced (Table 1), and spring seasonality accounted for 64% of all QFT positive results (Table 2). The measured IFN-*γ* production upon stimulation by* Mtb* antigens was often highest at the spring season time point among the serially tested HCWs with discordant results (Figure 1).

## 4. Discussion

Our GEE model accounted for paired data and showed that in HCWs with a spring season blood draw resulted in a 33 pg increase (0.825 IU) in the level of IFN-*γ* generated in response to TB antigen stimulation. A positive QFT result has an antigen result of 0.35 IU or greater (after subtracting the IFN-*γ* measured in the nil QFT tube). Factors also positively associated with an increase in antigenic response included IL-3 IL-1b, and IL-12p70. Those cytokines negatively associated with an increase in antigenic response were IL-7, GCSF, and IFN-*γ*.

### 4.1. Spring Season

In the present study, a significant seasonal relationship was found to be coupled with the frequency of QFT discordant results. The highest proportion of positive QFT results occurred during spring season blood draws in Houston which corresponds to when numerous environmental allergens are present in the air such as pollen from trees (Live Oak, Cedar, Ash, etc.), weeds (Ragweed, Sage, Sedge, etc.), grass, and mold spores. Previous studies have shown a seasonal relationship with whole blood cytokine levels produced after stimulation with bacterial proteins [7]. The measurable 0.825 IU (33 pg) increase in the circulating proinflammatory cytokine IFN-*γ* in the spring season increases TB antigen response to result in a positive QFT test resulting in discordant serial QFT results. A large percentage of false-positive QFT results seen in serial testing of HCWs may be accounted for by this seasonal response.

The increased levels of allergens in the air during the spring season in Houston can cause sensitized individuals to have allergic immunologic responses. IL-3 is commonly acknowledged as having growth factor-like activity, but during allergic responses it also carries out proinflammatory functions, such as stimulating basophils to secrete more IL-3 and express elevated levels of CD69 [11]. Therefore, during the spring season, individuals are more likely to have elevated IL-3 levels. However, IL-3 also activates the immune system when an individual has a severe TB infection. IL-3 has been seen to be upregulated in the lungs, lymph nodes, and spleen of TB infected rhesus monkeys [12].

Epithelial damage can occur when allergens enter the airways of a sensitized individual, and in response, the epithelial cells release danger signals, such as uric acid and ATP [13]. These danger signals stimulate macrophages to cleave pro-IL-1*β* molecules and secrete the mature cytokine. Mature IL-1*β* can enhance Th17 differentiation in the lungs by regulating IRF4 and ROR*γ*T expression during Th17 polarization and maintaining cytokine production of effector Th17 cells [13]. While IL-1*β* is a critical element to developing allergic inflammation, this cytokine is also essential in regulating the inflammatory response to* Mtb* infection.* In vivo *studies have shown that IL-1*β* and IL-6 are upregulated in the lungs of pulmonary TB patients perhaps due to the presence of lipoarabinomannan (LAM), a major cell wall component in* Mtb *bacteria [14].

In this study, the elevated levels of IL-1*β* and IL-3 in the discordant positive QFT results could have been due to a possible* Mtb *infection or due to allergens in the air during the spring season. It is noteworthy to discuss why IL-2 was not significantly elevated in the QFT discordant results. Increased immune activation due to antigenic stimuli leads to the increased expression of IL-2, but allergens and particulate matter, such as diesel exhaust particles (DEP), also increase the expression of IL-2. Allergens are unable to induce IL-2 expression on their own [15]. This study did not find any difference in IL-2 expression among the discordant QFT results; however, pollution is moderately present round the year in Houston because of the high volume of commuters and oil-refinery businesses. Therefore, we surmise that IL-2 is not responsible for the discordant results among the enrolled Houston HCWs.

Type 1 polarizing dendritic cells (DC1) produce bioactive IL-12p70 that facilitates the development of Th1 effector cells and IFN-*γ* production during an immune response [16]. In the present study, the amount of IL-12p70 was significantly elevated in samples producing QFT discordant results (*P* < 0.05). It is possible that IL-12p70 is being upregulated in patients due to factors other than a true* Mtb* infection, and this occurrence may cause patients to have false-positive QFT result.

There are several known factors that cause IL-12p70 levels to become elevated. Studies on allergic contact dermatitis using NiSO_4_ and 2,4-dinitrochlorobenzene (DNCB) have demonstrated that contact allergens can induce the production of IL-12p70 that stimulates the synthesis of IFN-*γ* from Th1 cells [17]. Contact allergens (due to pollution) present in the spring season in Houston may potentially cause elevated IL-12p70 levels in sensitive patients, and the number of discordant QFT results may increase. Numerous other factors can cause IL-12p70 levels to be elevated in an individual, which can lead to a corresponding increase of IFN-*γ* levels. If any of these additional factors occurred at the time of a blood draw for a QFT test, the results could potentially reflect a false-positive QFT result. Therefore, IL-12p70 levels may need to be considered to determine if a positive QFT result truly indicates an* Mtb *infection when a discordant test is seen during serial testing.

Limitations of this study include small sample size. This was an exploratory study, and only 9 of the 10 participants met the requirements for inclusion in the analysis. Furthermore, spring was arbitrarily defined as March–May. This does not take into account climate variability that may occur from year to year. Strengths of the study include the number of repeated measures included in the analysis and the large number of cytokines that were analyzed.

In conclusion, results from our study indicate that seasonal variation of cytokine levels in peripheral blood may lead to increased positive QFT results among patients with discordant serial results. These discordant positive results may represent false-positive QFT results induced by allergen stimulated production of cytokines. When using the QFT for serial testing for TBI, we recommend that seasonality be accounted for when evaluating diagnosis of TBI upon conversion from a negative to a positive QFT result in a short time period. Further research is needed to evaluate the effect of spring season on false-positive QFT test results.

## Figures and Tables

**Table 1:** Correlations between significant cytokines and chemokines with antigen induced IFN-*γ* level in QFT.

Covariants	Coefficients	*Z* value	*P* value	95% confidence interval
Interleukin-7 (IL-7)	−2.305	−1.99	0.047	[−4.58, −0.031]
Interferon-gamma (IFN-*γ*)	−0.534	−2.2	0.028	[−1.01, −0.059]
Interleukin-2 (IL-2)	−0.026	−0.03	0.972	[−1.527, 1.474]
Granulocyte colony-stimulating factor	−0.007	−2.1	0.036	[−0.014, −0.0004]
Soluble IL-2 receptor alpha (sIL-2R*α*)	−0.005	−0.05	0.963	[−0.206, 0.197]
Macrophage inflammatory protein 1*β* (MIP-1*β*)	−0.003	−0.32	0.746	[−0.02, 0.014]
Interleukin-6 (IL-6)	0.00004	0.21	0.835	[−0.003, 0.004]
Interleukin-1*β* (IL-1*β*)	0.044	2.35	0.019	[0.007,0.08]
Interleukin-15 (IL-15)	0.786	0.38	0.706	[−3.302, 4.874]
Interleukin-17 (IL-17)	1.183	1.36	0.173	[−0.519, 2.884]
Interleukin-12p70 (IL-12p70)	4.171	2.2	0.027	[0.463, 7.879]
Interleukin-3 (IL-3)	4.342	3.47	0.001	[1.889, 6.696]
Interleukin-5 (IL-5)	13.133	1.42	0.157	[−5.04, 31.306]
Spring season (March–May)	33.097	4.51	<0.001	[18.725, 47.47]

**Table 2:** QFT test results by season.

QFT result	Winter	Spring	Summer	Fall	Total
Negative	3 (13%)	**2 (9%)**	7 (32%)	10 (45%)	22
Positive	0 (0%)	**9 (64%)**	3 (21%)	2 (14%)	14
Total	3 (8%)	**11 (31%)**	10 (28%)	12 (33%)	36
